# Approximating the ideal observer for joint signal detection and estimation tasks by the use of Markov-Chain Monte Carlo with generative adversarial networks

**DOI:** 10.1117/1.JMI.12.5.051810

**Published:** 2025-10-21

**Authors:** Dan Li, Kaiyan Li, Weimin Zhou, Mark A. Anastasio

**Affiliations:** a University of Illinois at Urbana-Champaign, Department of Bioengineering, Urbana, Illinois, United States; b University of Arizona, Department of Radiology and Imaging Sciences, Tucson, Arizona, United States; c University of Arizona, Wyant College of Optical Sciences, Tucson, Arizona, United States

**Keywords:** Bayesian ideal observer, Markov-chain Monte Carlo, generative adversarial networks, joint detection and estimation tasks, estimation receiver operating characteristic curve

## Abstract

**Purpose:**

The Bayesian ideal observer (IO) is a special model observer that achieves the best possible performance on tasks that involve signal detection or discrimination. Although IOs are desired for optimizing and assessing imaging technologies, they remain difficult to compute. Previously, a hybrid method that combines deep learning (DL) with a Markov-Chain Monte Carlo (MCMC) method was proposed for estimating the IO test statistic for joint signal detection–estimation tasks. That method will be referred to as the hybrid MCMC method. However, the hybrid MCMC method was restricted to use cases that involved relatively simple stochastic background and signal models.

**Approach:**

The previously developed hybrid MCMC method is generalized by utilizing a framework that integrates deep generative modeling into the MCMC sampling process. This method employs a generative adversarial network (GAN) that is trained on object or signal ensembles to establish data-driven stochastic object and signal models, respectively, and will be referred to as the hybrid MCMC-GAN method. This circumvents the limitation of traditional MCMC methods and enables the estimation of the IO test statistic with consideration of broader classes of clinically relevant object and signal models.

**Results:**

The hybrid MCMC–GAN method was evaluated on two binary detection-estimation tasks in which the observer must detect a signal and estimate its amplitude if the signal is detected. First, a stylized signal-known-statistically (SKS) and background-known-exactly task was considered. A GAN was employed to establish a stochastic signal model, enabling direct comparison of our GAN-based IO approximation with a closed-form expression for the IO decision strategy. The results confirmed that the proposed method could accurately approximate the performance of the true IO. Next, an SKS and background-known-statistically (BKS) task was considered. Here, a GAN was employed to establish a stochastic object model that described anatomical variability in an ensemble of magnetic resonance (MR) brain images. This represented a setting where traditional MCMC methods are inapplicable. In this study, although a reference estimate of the true IO performance was unavailable, the hybrid MCMC–GAN produced area under the estimation receiver operating characteristic curve (AEROC) estimates that exceeded those of a sub-ideal observer that represented a lower bound for the IO performance.

**Conclusion:**

By combining GAN-based generative modeling with MCMC, the hybrid MCMC–GAN method extends a previously proposed IO approximation method to more general detection–estimation tasks. This provides a new capability to benchmark and optimize imaging-system performance through virtual imaging studies.

## 1 Introduction

Objective, task-based measures of image quality (IQ) are widely advocated for assessing and optimizing medical imaging systems.^1^^–^^4^ In preliminary studies of imaging technologies, model observers are sometimes utilized as a means for computing objective IQ measures.^1^^,^^3^^,^^5^^–^^8^ The ideal observer (IO) is a special model observer that has been advocated for the optimization of imaging hardware and data-acquisition designs.^2^^,^^9^^,^^10^ It can also reveal opportunities for advancing image processing algorithms to improve observer performance. This is because the IO implements the optimal decision strategy and exploits complete knowledge of task-relevant information in the provided image data.^2^ As such, the IO, by definition, defines the best possible performance of any observer.

Although theoretically optimal, the IO for detection tasks relies on computing the likelihood ratio, which requires exact knowledge of the conditional probability density functions of the image data under both signal-present and signal-absent hypotheses. In practice, these distributions are rarely known, making direct implementation of the IO infeasible. For signal detection tasks, Markov-Chain Monte Carlo (MCMC) methods have been introduced to approximate the IO,^10^ but these techniques are often limited to relatively simple stochastic object models. To overcome this restriction, a variety of learning-based approaches have been proposed that employ convolutional neural networks (CNNs) to approximate the IO test statistic for binary^11^ and multi-class detection^12^^,^^13^ or discrimination problems.

Detection-estimation tasks, in which a signal is first detected and then relevant parameters are estimated, arise in numerous applications.^14^^–^^17^ The IO for such tasks employs a modified likelihood ratio as its test statistic.^18^ Observer performance on binary detection tasks is commonly evaluated using the area under the receiver operating characteristic (ROC) curve (AUC). For detection–estimation tasks, the estimation receiver operating characteristic (EROC) curve^15^ is employed instead. The EROC curve characterizes the expected utility of the parameter estimates against the false positive fraction across decision thresholds, and the area under the EROC curve (AEROC) serves as a figure of merit.^15^ However, as with detection tasks, the IO performance for detection-estimation tasks is typically difficult to accurately estimate.

To enable IO approximation for detection-estimation tasks, a data-driven hybrid approach involving the MCMC method and CNNs was introduced by Li et al.^18^ This method will be referred to as the hybrid MCMC method. Although the hybrid MCMC method can be implemented with simple stochastic object and signal models, it cannot be applied to more general cases because the MCMC component requires sampling with tractable likelihoods and priors that are unavailable in these general settings.^19^

In this work, to address this, we leverage deep generative models^20^ to extend the domain of applicability of the previously developed method. This will enable the estimation of IO performance on detection-estimation tasks that involve more clinically relevant background and signal models. The proposed method is referred to as the hybrid MCMC–GAN method, because it replaces the standard MCMC method in the previously developed hybrid method with a generative adversarial network (GAN)-enabled MCMC method. First, we validate the hybrid MCMC–GAN method by use of a signal-known-statistically (SKS) and background-known-exactly (BKE) test case, where a GAN is employed to establish a stochastic signal model; this enables direct comparison of our GAN-based IO approximation against a known analytic solution. Next, we applied the hybrid MCMC–GAN method to a more clinically relevant SKS and background-known-statistically (BKS) task. In this case, a GAN is employed to establish a stochastic object model. Here, a reference estimate of IO performance is not available, so a suboptimal model observer is employed to establish a lower bound on IO performance.

The remainder of this paper is organized as follows. Section 2 contains a review of the salient aspects of joint detection-estimation tasks, the previously reported hybrid MCMC approach, and GANs. A description of the hybrid MCMC-GAN method is provided in Sec. 3. The numerical studies and the corresponding results are presented in Secs. 4 and 5. Finally, the article concludes in Sec. 6 with a discussion of the potential advantages and limitations of the hybrid MCMC-GAN method.

## 2 Background

### 2.1 Canonical Detection-estimation Task

In a binary detection-estimation task, an observer must first decide whether an image contains a signal or not, and if the signal is present, estimate the parameter of interest θ associated with the signal. The imaging process under these two hypotheses can be described as

$$H_{0}:\;g=Hf_{b}+n=b+n,\;H_{1}:\;g=H(f_{b}+f_{s}(\theta ))+n=b+s(\theta )+n.$$ (1)

Here, $g\in R^{M}$ denotes the measured image data, obtained by applying a deterministic imaging operator H to the object and adding noise $n\in R^{M}$. Depending on the considered task, the background $f_{b}$, the signal $f_{s}(\theta )$, or both, may be treated as random. A random background is characterized by a stochastic object model, which generates samples from the probability density function $p(f_{b})$. A random signal is characterized by a stochastic signal model, which generates samples from the conditional probability-density function $p(f_{s}|\theta )$; the signal parameter θ itself has prior p(θ). In this work, the term “stochastic object (or signal) model” refers to a model that can draw samples from the distributions of background objects or signals.

If $f_{b}$ is continuous, H is a continuous-to-discrete (C–D) mapping; if $f_{b}$ is approximated as an N dimensional vector, then $H\in R^{M\times N}$ is a discrete-to-discrete (D–D) mapping. The resulting background and signal images are $b\equiv Hf_{b}$ and $s(\theta )\equiv Hf_{s}(\theta )$, respectively.

In a binary detection-estimation task, a deterministic observer computes a test statistic T(g), mapping the image g to a real-valued scalar. Comparing T(g) to a threshold determines whether g satisfies $H_{0}$ or $H_{1}$. If the decision is signal-present ($H_{1}$), an estimate $\hat{\theta }(g)$ is obtained. An EROC curve is generated by plotting the expected utility of $\hat{\theta }(g)$ for true positives against the false positive fraction as the threshold varies. Typically, the utility function is constructed to yield high values when $\hat{\theta }(g)$ is close to the true θ.^15^

### 2.2 Ideal Observer for Detection-estimation Tasks

Let $u(\theta ,\hat{\theta })$ denote a utility function that quantifies the quality of an estimate $\hat{\theta }$ when the true parameter is θ. The corresponding IO for the detection–estimation task maximizes the AEROC, yielding the best achievable performance. The IO estimator of the parameter θ, denoted as $\hat{\theta }(g)$, is formed as^15^

θ^(g)=arg maxθ^∫pr(θ)Λ(g|θ)u(θ,θ^)dθ, (2)

where $\Lambda (g|\theta )=\frac{pr(g|\theta ,H_{1})}{pr(g|H_{0})}$ is the θ-conditional likelihood ratio and pr(θ) denotes the prior distribution of the parameter. Using this estimator $\hat{\theta }(g)$, the IO test statistic for the detection-estimation task can be expressed as^15^

T(g)=∫pr(θ)Λ(g|θ)u(θ,θ^(g))dθ. (3)

As described below, it is useful to express the IO statistic as the product of two terms^18^

T(g)=pr(g|H1)pr(g|H0)∫pr(θ|g,H1)u(θ^(g),θ)dθ=Λ(g)U(g). (4)

The first term $\Lambda (g)=\frac{pr(g|H_{1})}{pr(g|H_{0})}$ is the likelihood ratio, which corresponds to the IO test statistic for a pure binary detection task, rather than the detection-estimation task. The second term

U(g)=∫pr(θ|g,H1)u(θ^(g),θ)dθ, (5)

represents the utility-weighted posterior mean of the parameter of interest. Together, Λ(g) and U(g) characterize the IO test statistic for detection-estimation tasks.

The IO test statistic for detection-estimation tasks is generally analytically intractable and difficult to compute. For simple stochastic object and signal models, a hybrid Markov-chain Monte Carlo method, originally introduced by Li et al.^18^ can be employed. In this framework, the likelihood ratio Λ(g) and the estimate $\hat{\theta }(g)$ are approximated using CNN, whereas the utility-weighted posterior mean U(g) is approximated by MCMC sampling, hence the term “hybrid MCMC,” as described next.

#### 2.2.1 Approximating the likelihood ratio and parameter estimate using CNNs

The likelihood ratio for the pure binary detection task, Λ(g), can be approximated by training a CNN with a cross-entropy loss to estimate the posterior probability $Pr(H_{1}|g)$,^11^ which is a monotonic function of Λ(g). In addition, the ideal parameter estimate can be approximated by training a CNN to maximize the expected utility U(g) over all signal-present images g.^18^ The loss functions for estimating Λ(g) and the ideal parameters $\hat{\theta }$ are specified as^18^

LDetection(w1)=−12J∑j=12Jp(yi|gi)log(p(y^i|gi,w1)), (6)

and

LEstimation(w2)=−1J∑j=1Ju(θ^j(w2),θj), (7)

where $w_{1}$ and $w_{2}$ denote the CNN’s weights for the detection and estimation sub-networks, respectively. For the detection sub-network, the loss function used is the cross-entropy loss.

#### 2.2.2 Approximating the utility-weighted posterior mean using MCMC

For simple object models, the utility-weighted posterior mean U(g) can be estimated using MCMC methods. In particular, the quantity U(g) from Eq. (5) may be approximated via Monte Carlo simulation as

$$\hat{U}(g)=\frac{1}{J}\sum_{j=1}^{J}u(\hat{\theta }(g),\theta_{j}),$$ (8)

where $\theta^{j}$ is sampled from the posterior $p(\theta |g,H_{1})$. A Markov chain, employing a proposal density $q(\theta^{j}|\theta^{j-1})$, is used to generate the samples $\theta^{j}$.

Given $\theta^{j}$, a candidate parameter $\theta^{*}$ is drawn from the proposal density $q(\theta^{*}|\theta^{j})$ and accepted into the Markov chain with probability^18^

$$p_{a}(\theta^{*}|\theta^{j},g)=\min\{1,\frac{p(g|s(\theta^{*}),H_{1})p_{\theta }(\theta^{*})q(\theta^{j}|\theta^{*})}{p(g|s(\theta^{j}),H_{1})p_{\theta }(\theta^{j})q(\theta^{*}|\theta^{j})}\},$$ (9)

where $p_{\theta }$ is the prior on θ. The signal image realizations $s(\theta^{j})$ and $s(\theta^{*})$ correspond to parameter estimates $\theta^{j}$ and $\theta^{*}$, respectively. If the candidate is accepted, $\theta^{j+1}\equiv \theta^{*}$; otherwise, $\theta^{j+1}\equiv \theta^{j}$. For an SKS/BKE task where the prior distribution of θ is known, the term $p(g|s(\theta ),H_{1})$ in Eq. (9) is determined by the noise distribution, because θ defines the signal image s.

For an SKE/BKS task where background variability is introduced through a stochastic object model parameterized by α, the corresponding acceptance probability is given by^18^

$$p_{a}(\theta^{*},\alpha^{*}|\theta^{j},\alpha^{j},g)=\min\{1,\frac{p(g|s(\theta^{*}),b(\alpha^{*}),H_{1})p_{\theta }(\theta^{*})p_{\alpha }(\alpha^{*})q_{1}(\theta^{*}|\theta^{j})q_{2}(\alpha^{*}|\alpha^{j})}{p(g|s(\theta^{j}),b(\alpha^{j}),H_{1})p_{\theta }(\theta^{j})p_{\alpha }(\alpha^{j})q_{1}(\theta^{j}|\theta^{*})q_{2}(\alpha^{j}|\alpha^{*})}\}.$$ (10)

Here, $q_{1}$ and $q_{2}$ denote the proposal density functions for the candidate parameters $\theta^{*}$ and $\alpha^{*}$, respectively. The quantity $p_{\alpha }$ represents the prior probability distribution for α. Because α determines the background image b in Eq. (1), the term $p(g|s(\theta^{*}),b(\alpha^{*}),H_{1})$ is similarly determined by the distribution of the noise n.

### 2.3 Extending MCMC by the Use of Generative Adversarial Networks

Conventional MCMC-based IO approximation methods require explicit stochastic object or signal models (analytic priors) and thus become inapplicable when such parameterized models are unavailable. To address this gap, GANs can be employed to represent stochastic object or signal models.^12^ Zhou et al.^12^ demonstrated and validated this approach by integrating a GAN-based stochastic object model into an MCMC sampler to approximate the IO test statistic for binary detection tasks.

GANs achieve this implicit modeling through an adversarial process using two deep neural networks. A generator G maps low-dimensional latent vectors z to synthetic images, whereas a discriminator D assesses the realism of each sample. During training, G and D engage in the two-player minimax game.^20^ At the global optimum of the minimax game, the generator’s output distribution matches that of the training data. The trained generator can thus provide an implicit stochastic model parameterized by the latent vector z.^12^

Compared with diffusion models that require dozens to hundreds of forward passes to generate an image, a GAN requires only a single forward pass. Therefore, inference times of GANs are generally 1 to 2 orders of magnitude smaller than diffusion models. This enables a faster sample generation in an MCMC procedure.^21^

Depending on the task, the generator may be trained in either object-space or image-space. In object-space training, G is trained on discretized background objects $f_{b}\in R^{N}$ obtained from a procedure-based stochastic object model—such as VICTRE breast phantoms or other simulation-based ensembles. Once trained, the background image can be generated as b^(z)=HG(z;wG), where $H\in R^{M\times N}$ is the imaging operator. Alternatively, if the generator G is trained directly on measured background images $b\in R^{M}$ (image-space training), then b^(z)=G(z;wG). Similarly, for stochastic signal models, if the GAN is trained directly on discretized signals $f_{s}\in R^{N}$, we have s^(z)=HG(z;wG). When GAN is trained on images of the signals $s\in R^{M}$, then s^(z)=G(z;wG).

In addition, in scenarios where noisy experimental images from a well-characterized imaging system are available to define a stochastic object model, an AmbientGAN framework can directly learn the object distribution from these measurements.^22^^,^^23^

### 2.4 Supervised Learning-based Sub-ideal Observer

One can avoid the need for the MCMC method using the likelihood ratio Λ(g) in Eq. (4) directly as a sub-ideal test statistic for joint detection–estimation tasks. In this approach, the estimation results no longer affect the detection performance. The corresponding test statistic and estimator are defined by

$$T_{\text{Sub}}(g)=\frac{\mathrm{pr}(g|H_{1})}{\mathrm{pr}(g|H_{0})}=\Lambda (g),$$ (11)

and

θ^Sub(g)=arg maxθ^∫pθ(θ)Λ(g|θ)u(θ,θ^)dθ. (12)

Here, $T_{\text{Sub}}(g)$ and θ^Sub(g) can be learned using deep learning approaches that minimize the loss functions in Eqs. (6) and (7),^18^ respectively.

## 3 Methods

As outlined in Sec. 2.2, the IO test statistic T(g) can be decomposed into two components: the likelihood ratio Λ(g) and the utility-weighted posterior mean U(g), as indicated in Eq. (4). The likelihood ratio Λ(g) can be approximated alongside the ideal estimate $\hat{\theta }(g)$ by minimizing the CNN loss functions in Eqs. (6) and (7). However, computing U(g) requires sampling from a stochastic signal or object model—a task generally intractable without an explicit stochastic model. This limitation was addressed by incorporating GAN-based implicit models into the MCMC sampler, following the approach first proposed by Zhou et al.^12^ In the next two subsections, the conventional MCMC method is generalized to use GAN-based signal or object models. We demonstrate the method on two canonical tasks: SKS/BKE and SKS/BKS.

### 3.1 Utility-Weighted Posterior Mean for a SKS/BKE Task

Consider an SKS/BKE task lacking an explicit stochastic signal model, such as VICTRE Lesion Models or other simulation-based ensembles. If a GAN is trained on samples from the underlying signal distribution, the generated signal image parameterized by the latent vector z is s^(z)=HG(z;wG). If, instead, the GAN is trained directly on signal images $s\in R^{M}$, the synthesis becomes s^(z)=G(z;wG).

The parameter of interest θ(z) associated with $\hat{s}(z)$, as defined in Eq. (3), can be obtained from a trained CNN estimator or, when measurement noise is not added, via an analytic expression. Because both the signal parameter θ and the latent vector z uniquely determine the signal, substituting z for θ in Eq. (5) yields the utility-weighted posterior mean

U(g)=∫p(z|g,H1)u(θ^(g),θ(z))dz=∫p(g|s^(z),H1)pz(z)Pr(g|H1)u(θ^(g),θ(z))dz. (13)

An unbiased Monte-Carlo estimate, based on J samples $\{z^{j}{\}}_{j=1}^{J}$, can be computed as

$$\hat{U}(g)=\frac{1}{J}\sum_{j=1}^{J}u(\hat{\theta }(g),\theta (z^{j})).$$ (14)

Here, the samples $z^{j}∼p(z|g,H_{1})$ are obtained via MCMC sampling with acceptance probability

pa(z*|zj,g)=min{1,p(g|s^(z*),H1)pz(z*)q(zj|z*)p(g|s^(zj),H1)pz(zj)q(z*|zj)}. (15)

In this study, the pre-conditioned Crank–Nicolson (pCN) proposal for the proposal density q(·|·) was adopted. The prior $p_{z}$ is available in closed form since $z∼N(0,I_{k})$. Given a current state $z^{j}$ and step size β

$$z^{*}=\sqrt{1-\beta^{2}}z^{j}+\beta \xi ,\;\xi ∼N(0,I_{k}).$$ (16)

With this proposal being prior-invariant

$$p_{z}(z^{*})q(z^{j}|z^{*})=p_{z}(z^{j})q(z^{*}|z^{j}),$$ (17)

the acceptance probability simplifies to

pa(z*|zj,g)=min{1,p(g|s^(z*),H1)p(g|s^(zj),H1)}. (18)

If accepted, $z^{j+1}=z^{*}$; otherwise, $z^{j+1}=z^{j}$.

### 3.2 Utility-weighted Posterior Mean for a SKS/BKS Task

Consider an SKS/BKS task for which an explicit stochastic object model is lacking, whereas an ensemble of discretized objects is available, such as VICTRE breast phantoms or other simulation-based ensembles. If a GAN is trained on samples from the underlying object distribution, the generated object image parameterized by a latent vector z is b^(z)=HG(z;wG). If, instead, the GAN is trained directly on measured object images $b\in R^{M}$, the synthesis becomes b^(z)=G(z;wG).

With this GAN-based stochastic object model, the utility-weighted posterior mean given by Eq. (5) can be expressed as

U(g)=∫p(θ|g,H1)μ(θ^(g),θ)dθ=∬p(g|s(θ),b(z),H1)pz(z)pθ(θ)μ(θ^(g),θ)pr(g|H1)dz dθ. (19)

An unbiased Monte-Carlo estimate is given by

U^(g)=1J∑j=1Ju(θ^(g),θj). (20)

Here, the samples $\left(\theta^{j},z^{j}\right)$ are generated by the MCMC sampling method: at each iteration j, a candidate $\left(\theta^{*},z^{*}\right)$ is drawn from the proposal $q_{1}(\theta^{*}|\theta^{j})q_{2}(z^{*}|z^{j})$ and accepted with probability

$$p_{a}(\theta^{*},z^{*}|\theta^{j},z^{j},g)=\min\{1,\frac{p(g|s(\theta^{*}),b(z^{*}),H_{1})p_{\theta }(\theta^{*})p_{z}(z^{*})q_{1}(\theta^{*}|\theta^{j})q_{2}(z^{*}|z^{j})}{p(g|s(\theta^{j}),b(z^{j}),H_{1})p_{\theta }(\theta^{j})p_{z}(z^{j})q_{1}(\theta^{j}|\theta^{*})q_{2}(z^{j}|z^{*})}\}.$$ (21)

When using the pre-conditioned Crank-Nicolson (pCN) proposal for z, similar to what was described in Sec. 3.1, the ratio in the acceptance probability $p_{a}$ simplifies, because $\frac{p_{z}(z^{*})q_{2}(z^{j}|z^{*})}{p_{z}(z^{j})q_{2}(z^{*}|z^{j})}=1$. Similarly, a symmetric Gaussian proposal for θ yields $\frac{q_{1}(\theta^{j}|\theta^{*})}{q_{1}(\theta^{*}|\theta^{j})}=1$, and a uniform prior on θ implies $p_{\theta }(\theta^{*})/p_{\theta }(\theta^{j})=1$. Consequently, the acceptance probability reduces to

$$p_{a}(\theta^{*},z^{*}|\theta^{j},z^{j},g)=\min\{1,\frac{p(g|s(\theta^{*}),b(z^{*}),H_{1})}{p(g|s(\theta^{j}),b(z^{j}),H_{1})}\}.$$ (22)

## 4 Numerical Studies

Computer simulation studies were performed to validate and demonstrate the proposed hybrid MCMC-GAN method for approximating the IO for joint detection–estimation tasks. Two studies were considered.

First, to validate the method against a known analytic solution, a SKS/BKE task was considered. In this study, signals were generated both analytically and with a GAN-based stochastic signal model. The IO performance estimate yielded by the hybrid MCMC–GAN method was compared with the corresponding analytic IO solution. Next, an SKS/BKS task was considered, where the hybrid MCMC–GAN method was applied to a task that involved MRI brain images that cannot be described by any known stochastic object model, making conventional MCMC approaches infeasible. In both settings, observer performance was characterized by EROC curves and summarized by AEROC values. Details regarding each study are presented below.

### 4.1 SKS/BKE Task with a GAN-Based Signal Model

In the SKS/BKE study, a binary detection–estimation task was considered that involved at most one signal per image. The background was known exactly, and the signal’s location and shape were fixed, whereas its amplitude $A_{s}$ was assumed random. The observer must decide between $H_{0}$ (signal absent) and $H_{1}$ (signal present); if $H_{1}$ is chosen, it produces an estimate ${\hat{A}}_{s}$ of the true amplitude $A_{s}$.

**Fig. 1:**
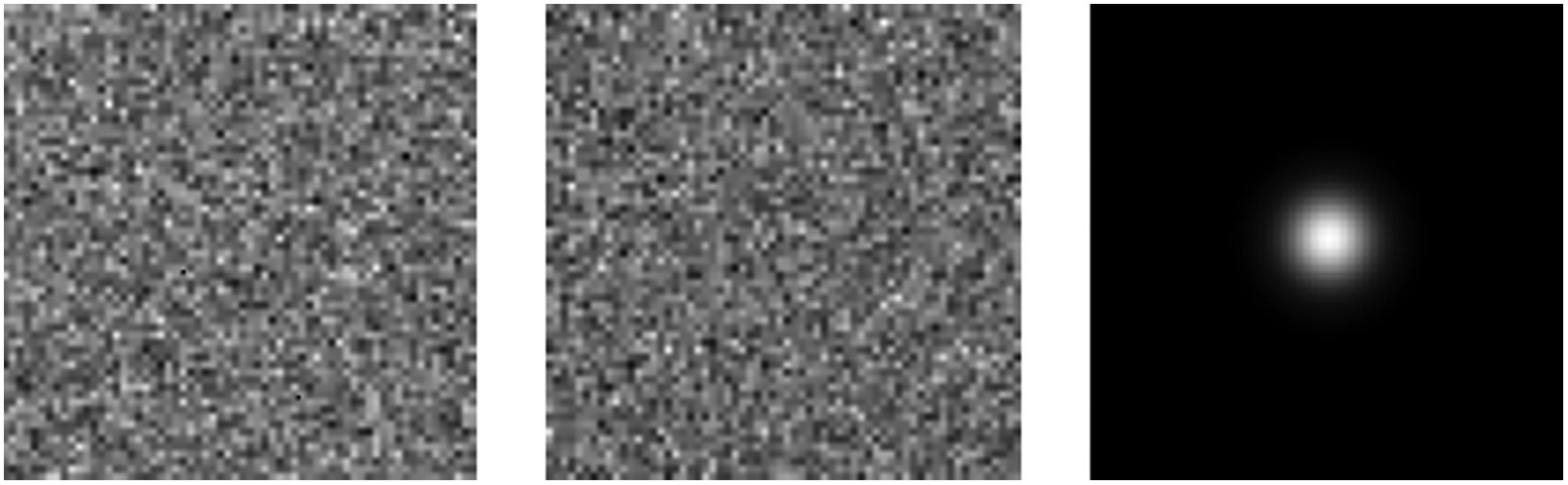
Left: noise (signal-absent image), middle: signal-present image, and right: signal image.

The measured image g, background b (set to 0 without loss of generality), and additive Gaussian noise n were all represented as 64×64 arrays. The signal was defined as a Gaussian function $f_{s}(r)=A_{s}\;\exp(-\frac{\left(r-r_{s}{)}^{T}(r-r_{s}\right)}{2w_{s}^{2}})$, where $w_{s}=1$ and $r_{s}=(32,32{)}^{T}$. Signal amplitudes were drawn from a normal distribution, i.e., $A_{s}∼N(\mu_{A},\sigma_{A}^{2})$, with $\mu_{A}=9$, $\sigma_{A}=4$. The imaging system was described by the point response function (PRF) $h_{m}(r)=\frac{h}{2\pi w_{m}^{2}}\;\exp(-\frac{\left(r-r_{m}{)}^{T}(r-r_{m}\right)}{2w_{m}^{2}})$,^2^ where h=16 and $w_{m}=3.87$. The m’th component of the signal image s was given by $s_{m}=\frac{A_{s}hw_{s}^{2}}{w_{m}^{2}+w_{s}^{2}}\;\exp[-\frac{\left(r_{m}-r_{s}{)}^{T}(r_{m}-r_{s}\right)}{2(w_{m}^{2}+w_{s}^{2})}]$. Measurement noise was modeled as i.i.d. Gaussian: $n∼N(0,\sigma_{n}^{2})$, with $\sigma_{n}=40$. Observer performance was quantified by an EROC curve based on the Gaussian utility u(A^s,As)=exp[−(A^s−As)2/(2σu2)], with $\sigma_{u}=3$. One realization of the signal image s, noise n, and the corresponding signal-present noisy measurement g are shown in Fig. 1.

A progressive GAN^24^ was trained on 100,000 simulated signal realizations. Once trained, signal images were synthesized as s^(z)=G(z). The utility-weighted posterior mean U(g) was approximated according to Eq. (14) by sampling latent vectors $z^{j}$ via the pCN algorithm with the acceptance probability in Eq. (18).

The performance of the IO for the joint detection–estimation task was approximated via the proposed hybrid MCMC–GAN method and was compared with the analytic IO whose decision rule is given by^25^

A^I(g)=σA2srefTg+σn2μAσn2+σA2‖sref‖2,TI(g)=μAsrefTg+σA22σn2(srefTg)2, (23a)

$$[s_{\mathrm{ref}}{]}_{m}=\frac{hw_{s}^{2}}{w_{m}^{2}+w_{s}^{2}}\;\exp[-\frac{\|r_{m}-r_{s}{\|}^{2}}{2(w_{m}^{2}+w_{s}^{2})}].$$ (23b)

Each observer was evaluated on 400 signal-present and 400 signal-absent images, and detection–estimation performance was quantified using EROC curves.

### 4.2 SKS/BKS Task with a GAN-Based Object Model

**Fig. 2:**
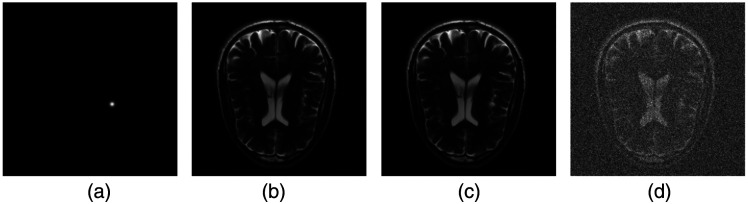
From left to right: (a) the signal template s(r), (b) GAN-synthesized background image $\hat{b}(z)$, (c) noise-free composite $s+\hat{b}$, and (d) the composite with additive Gaussian noise.

In the SKS/BKS study, clinical brain magnetic resonance (MR) images served as stochastic backgrounds, described by a GAN-based stochastic object model. A binary detection–estimation task was considered in which the signal’s location and shape were fixed, whereas its amplitude $A_{s}$ was considered random and sampled from the uniform distribution U(0.05,0.15). The observer must decide between $H_{0}$ (signal absent) and $H_{1}$ (signal present); if $H_{1}$ is chosen, it produces an estimate ${\hat{A}}_{s}$ of the true amplitude $A_{s}$. The signal image is defined by $s(r)=A_{s}\;\exp[-(r-r_{s}{)}^{T}(r-r_{s})/(2w_{s}^{2})]$, with $w_{s}=3$ and $r_{s}=(150,160{)}^{T}$. Measurement noise n was modeled as i.i.d. Gaussian, $n∼N(0,\sigma_{n}^{2})$ with $\sigma_{n}=0.01$. Observer performance was assessed via the EROC curve using the Gaussian utility $u({\hat{A}}_{s},A_{s})=\exp[-({\hat{A}}_{s}-A_{s}{)}^{2}/(2\sigma_{u}^{2})]$, with $\sigma_{u}=0.05$. One realization of the signal image s, the GAN-generated background $\hat{b}$, and the corresponding noise-free and noisy signal-present images g are shown in Fig. 2.

The background images were defined as T2-weighted axial brain MR images (256×256  pixels) from the fastMRI dataset.^26^ This dataset comprises 20,000 images (10 per subject) from 2000 subjects, all normalized to the interval [0, 1]. A progressive GAN was trained on these images to establish a stochastic object model for use in the hybrid MCMC-GAN method. The utility-weighted posterior mean U(g) in Eq. (19) was approximated according to Eq. (20) via MCMC sampling through a GAN-based object model.

It should be noted that, because it involves a traditional MCMC sampling method, the hybrid MCMC method developed by Li et al.^18^ was not applicable in this study. In addition, the IO decision strategy is not analytically tractable for this task. As such, no alternative IO methods were available to serve as references. Instead, the hybrid MCMC-GAN (with a GAN-based signal model and pCN sampling) was compared with the sub-ideal observer described in Sec. 2.4, which provides a lower bound on IO performance. Each observer was evaluated on 300 signal-present and 300 signal-absent images, and detection–estimation performance was quantified using EROC curves.

### 4.3 Hybrid MCMC-GAN Implementation Details

The original ProGAN architecture of Karras et al.^24^ was adopted, which begins at a 4×4 image resolution and employs a 256-dimensional latent space. Training was performed with the publicly available ProGAN code^27^ and the Adam optimizer.^28^ A single NVIDIA RTX 4500 GPU was employed for training and inference.

After convergence, the Metropolis–Hastings sampling was performed with the pre-conditioned Crank–Nicolson (pCN) proposal^29^ to generate latent vectors z and θ for computing the utility-weighted posterior mean U(g) and test statistic T(g). The corresponding proposal densities and acceptance probabilities are specified in Sec. 3.

## 5 Results

### 5.1 SKS/BKE Task with a GAN-Based Signal Model

**Fig. 3:**
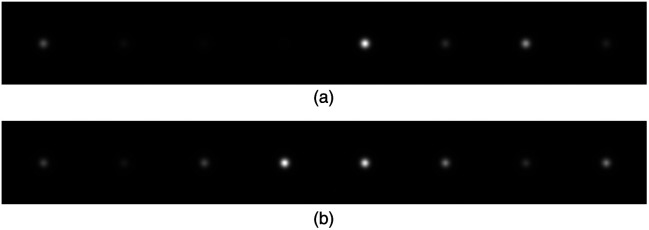
Comparison of analytically generated (“real”) signals and GAN-generated (“fake”) signals. (a) Real: signals generated analytically. (b) Fake: signals generated by GAN.

**Fig. 4:**
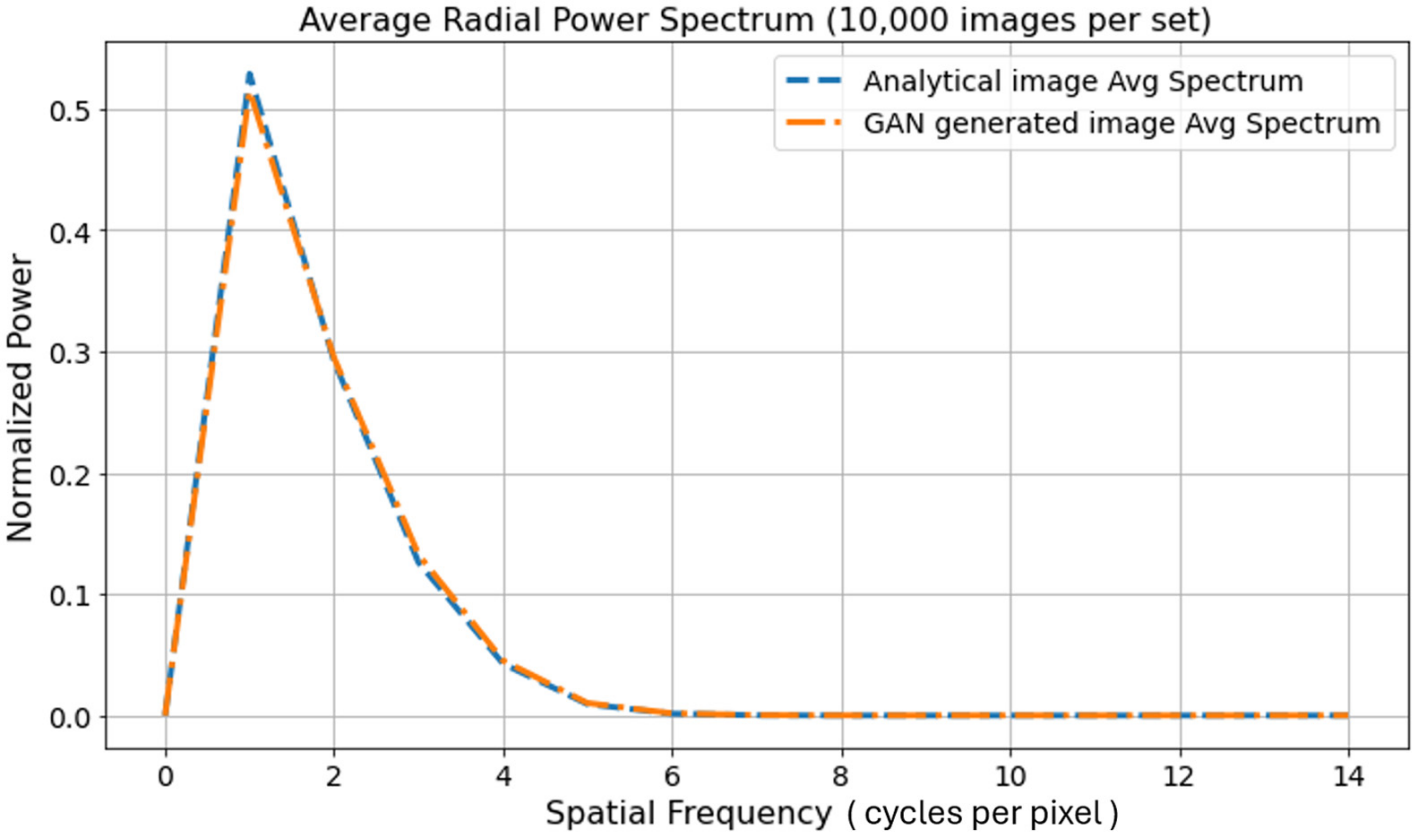
Normalized radial power spectra of real and GAN-generated signal images (64×64), each averaged over 10,000 samples. The maximum radial index (Euclidean distance from the Fourier center) is 46, but only the first 15 frequency bins are shown because the spectrum is zero beyond that point. The image mean is removed (no DC component). The power spectra of the real and GAN-generated signal images match closely, indicating that the GAN captured certain second-order statistics of the real signal images.

Examples of ground-truth and ProGAN-generated signals are shown in Fig. 3, which appear visually indistinguishable. The radially averaged power spectra of 10,000 real and 10,000 ProGAN signals were also computed. As shown in Fig. 4, the two curves were nearly identical. Additional comments regarding GAN validations are provided in Sec. 6.

**Fig. 5:**
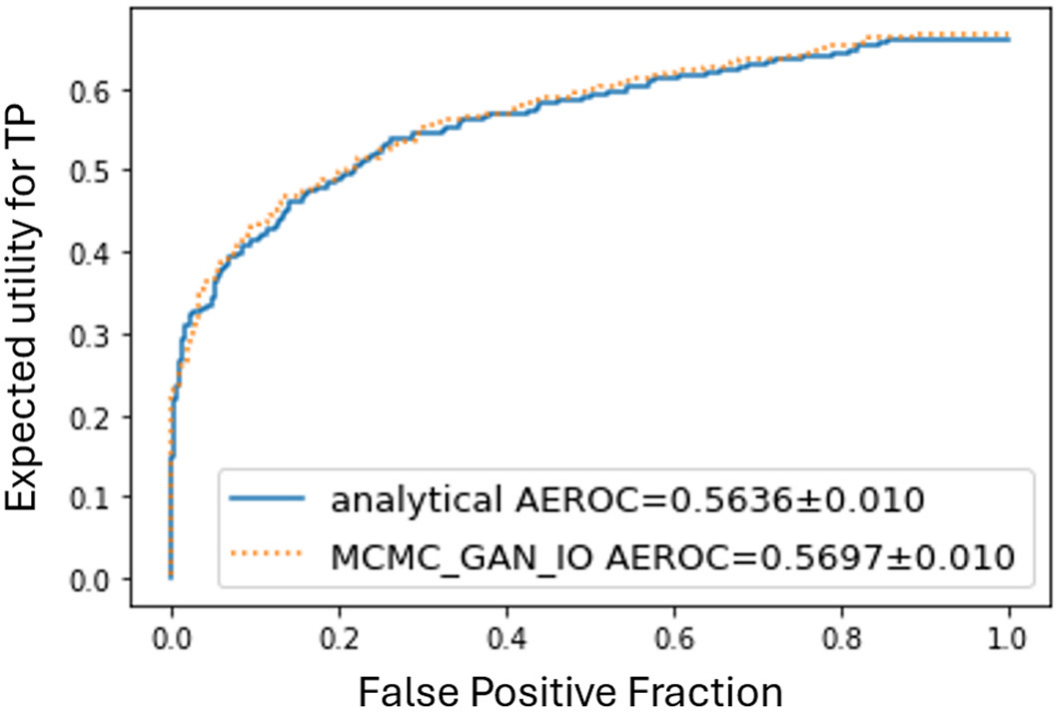
EROC curves comparing the analytic IO (solid blue) with the hybrid MCMC-GAN-based IO (dotted green) for the joint task of signal detection and subsequent amplitude estimation (SKS/BKE). The corresponding AEROC values are 0.5636 and 0.5697, respectively. This difference is within the confidence bounds; therefore, the two observers were statistically equivalent.

Figure 5 compares the EROC curves for the analytic IO (blue solid), the conventional MCMC IO (yellow dashed), and the hybrid MCMC-GAN–based IO (green dotted). The corresponding AEROC scores were 0.5636 and 0.5697. Given an estimated uncertainty of ±0.01 (95% confidence), this difference is within the confidence bounds; therefore, the two observers are statistically equivalent.

### 5.2 SKS/BKS Task with a GAN-Based Object Model

**Fig. 6:**
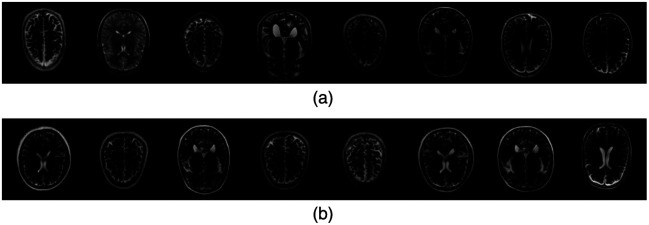
Comparison of real and GAN-generated MRI images. The GAN produced visually plausible images. (a) Real: clinical MRI images. (b) Fake: MRI images generated by the GAN.

**Fig. 7:**
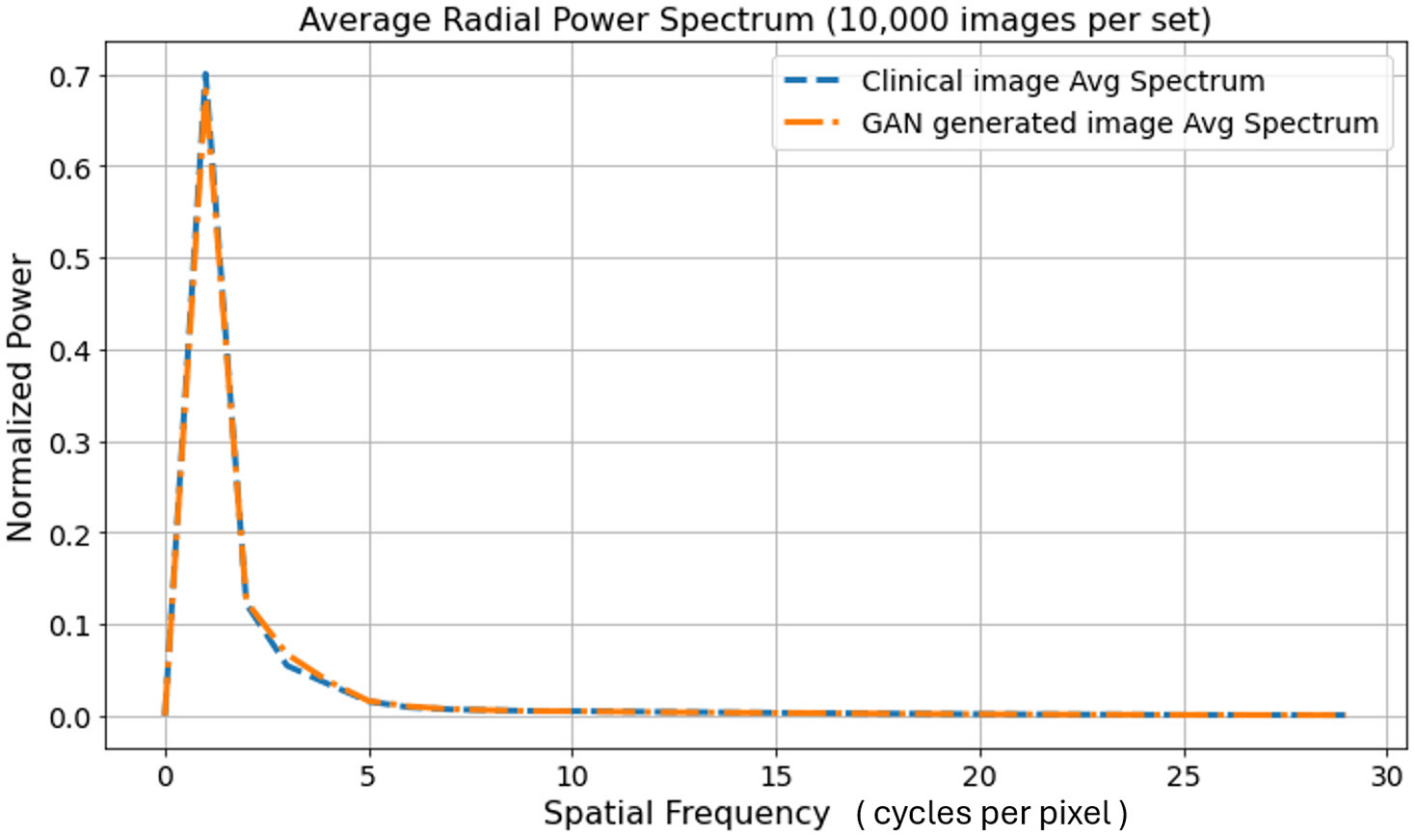
Normalized radial power spectra of real and GAN-generated MRI images (256×256), each averaged over 10,000 samples. The maximum radial index (Euclidean distance from the Fourier center) is 182, but only the first 30 frequency bins are shown because the spectrum is zero beyond that point. The image mean is removed (no DC component). The power spectra of the real and GAN-generated background object images match closely, indicating that the GAN captures the second-order statistics of the real background object images.

Examples of ground-truth and ProGAN-generated background images are shown in Fig. 6, which appear to be visually comparable. To evaluate how well the ProGAN-generated MRI images capture the statistical properties of the training data, we also computed the radially averaged power spectra of 10,000 real and 10,000 ProGAN images. As shown in Fig. 7, the two curves are quite similar. This similarity suggests that the ProGAN is able to replicate certain characteristics of the spatial frequency content present in the training data, as reflected in the radial power spectrum analysis. We acknowledge, however, that power spectrum analysis captures only specific second-order statistical properties of the images and does not fully characterize image realism or diagnostic fidelity. Additional methods—such as structural similarity indices, feature-based discriminants, or higher-order texture analyses—might be more sensitive to subtle discrepancies between synthetic and real images.^30^ Although such investigations are beyond the scope of this work, future studies may consider more comprehensive statistical comparisons, particularly in clinically sensitive applications where nuanced differences may affect observer performance or diagnostic outcomes.

**Fig. 8:**
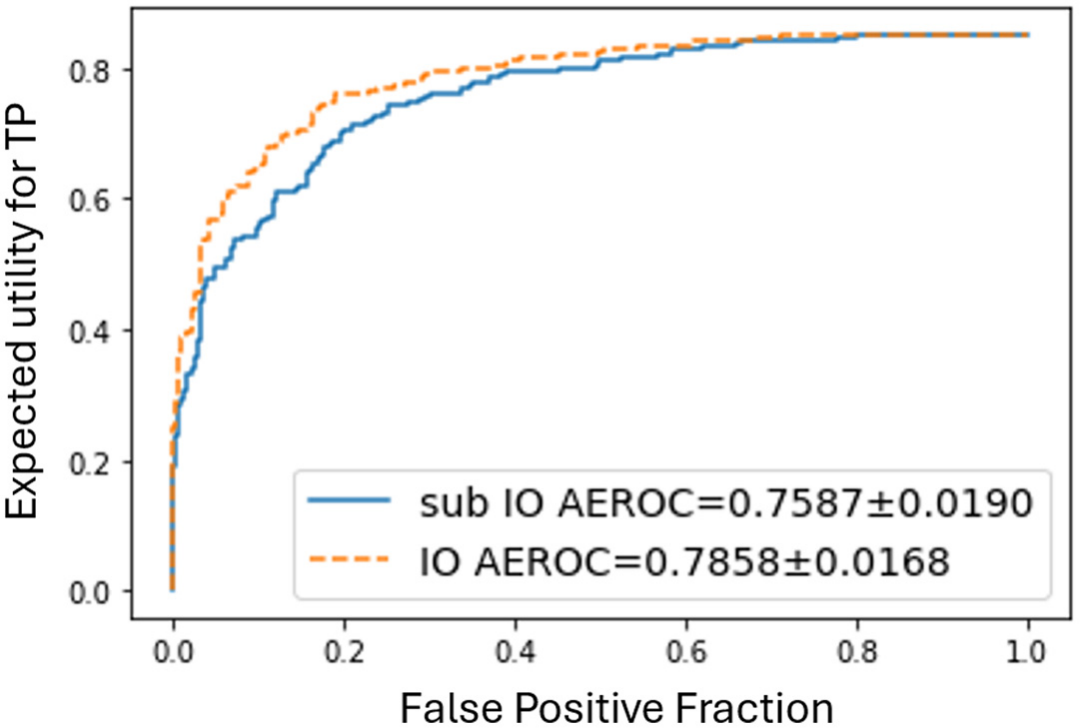
EROC curves comparing the hybrid MCMC–GAN-based IO (yellow dashed) with the sub-ideal observer (solid blue) for the joint task of signal detection and subsequent amplitude estimation (SKS/BKS). The hybrid MCMC-GAN observer achieves an AEROC of 0.7858 versus 0.7587 for the sub-ideal observer. The results demonstrate the superiority of the hybrid MCMC–GAN method in estimating the IO AEROC compared with a sub-ideal observer.

Figure 8 presents the EROC curves for the IO approximation based on hybrid MCMC-GAN (yellow dashed) and the sub-ideal observer (blue solid), the latter employing only the detection likelihood ratio Λ(g) as its test statistic (Sec. 2.4). The corresponding AEROC values are 0.7858 for the hybrid MCMC–GAN observer and 0.7587 for the sub-ideal observer. As required by an IO, the hybrid MCMC–GAN method produced an EROC curve that was nowhere lower than the one corresponding to the sub-ideal observer.

## 6 Discussion

In medical imaging, joint signal detection–estimation tasks can be evaluated using the EROC curve. However, directly computing the IO for such tasks is generally intractable. Li et al.^18^ addressed this challenge by combining MCMC sampling with CNNs to approximate the IO when relatively simple stochastic object or signal models are involved. However, because it relies on the traditional MCMC method, this method does not extend readily to more general detection–estimation scenarios for which closed-form descriptions of the required stochastic object or signal models are unavailable.

In this work, a hybrid MCMC–GAN method was introduced that replaces the standard MCMC method with a GAN-augmented sampling strategy. Deep generative models, particularly GANs, offer a flexible data-driven approach to establish stochastic object or signal models. They have seen increasing application in radiology for tasks such as synthesizing rare pathologies, augmenting limited datasets, and enhancing the performance of CNNs in diagnostic classification settings.^31^^,^^32^ In our study, we primarily leverage the GAN’s capability to learn the underlying distribution from which the training samples are drawn. The hybrid MCMC–GAN method was validated and explored in two numerical studies. First, a simple SKS/BKE task was considered with a GAN-based stochastic signal model. The proposed method yielded AEROC values that were comparable to those produced by the analytically known IO test statistic, serving as a validation of the proposed method. Second, a detection–estimation problem was considered that utilized clinical brain MR backgrounds—an application for which no analytic stochastic object model exists—and demonstrated that the hybrid MCMC-GAN method achieved a higher AEROC than a sub-ideal observer. This suggests that the hybrid MCMC–GAN method has the potential to extend IO approximations to more general detection–estimation problems.

Several aspects of the hybrid MCMC–GAN framework merit further consideration. First, no standardized criterion currently exists to ensure that a trained GAN accurately reproduces the true stochastic distribution of objects or signals, leaving open the question of whether the generated samples capture all relevant image statistics.^30^^,^^33^ Second, our proof-of-concept implementation varies only a single signal parameter (amplitude) and uses GANs to model either a stochastic signal or a stochastic object—but not both concurrently. By contrast, clinical detection–estimation tasks typically involve simultaneous variations in multiple parameters (e.g., lesion size, shape, and contrast). Therefore, it will be valuable to expand the framework to support the concurrent variation of several parameters with combined signal-object stochastic modeling. Moreover, the hybrid MCMC–GAN method relies on sufficiently large training datasets to establish both the GAN-based object/signal models and the hybrid CNN estimator; future work should quantify the impact of limited training data on performance. Finally, due to the modular nature of the proposed method, alternative deep generative networks can be readily explored to replace the use of GANs. In this case, however, the deep generative model should be compliant with the computational demands of the MCMC method. Many of the currently available diffusion models,^34^^,^^35^ for example, do not satisfy this requirement due to their relatively slow inference times.

## Data Availability

The data and code used in this study are either publicly available through links provided in the relevant citations or can be readily reproduced using standard procedures described in Sec. 3.
